# Development of Non-Invasive Continuous Glucose Prediction Models Using Multi-Modal Wearable Sensors in Free-Living Conditions

**DOI:** 10.3390/s25103207

**Published:** 2025-05-20

**Authors:** Thilini S. Karunarathna, Zilu Liang

**Affiliations:** 1 Ubiquitous and Personal Computing Laboratory, Kyoto University of Advanced Science (KUAS), Kyoto 615-8577, Japan; 2 Institute of Industrial Science, The University of Tokyo, Tokyo 153-8505, Japan

**Keywords:** continuous glucose monitoring, non-invasive, wearable sensors, multi-modal, machine learning

## Abstract

Continuous monitoring of glucose levels is important for diabetes management and prevention. While traditional glucose monitoring methods are often invasive and expensive, recent approaches using machine learning (ML) models have explored non-invasive alternatives—but many still depend on manually logged food intake and activity, which is burdensome and impractical for everyday use. In this study, we propose a novel approach that eliminates the need for manual input by utilizing only passively collected, automatically recorded multi-modal data from non-invasive wearable sensors. This enables practical and continuous glucose prediction in real-world, free-living environments. We used the BIG IDEAs Lab Glycemic Variability and Wearable Device Data (BIGIDEAs) dataset, which includes approximately 26,000 CGM readings, simultaneous ly collected wearable data, and demographic information. A total of 236 features encompassing physiological, behavioral, circadian, and demographic factors were constructed. Feature selection was conducted using random-forest-based importance analysis to select the most relevant features for model training. We evaluated the effectiveness of various ML regression techniques, including linear regression, ridge regression, random forest regression, and XGBoost regression, in terms of prediction and clinical accuracy. Biological sex, circadian rhythm, behavioral features, and tonic features of electrodermal activity (EDA) emerged as key predictors of glucose levels. Tree-based models outperformed linear models in both prediction and clinical accuracy. The XGBoost (XR) model performed best, achieving an R-squared of 0.73, an RMSE of 11.9 mg/dL, an NRMSE of 0.52 mg/dL, a MARD of 7.1%, and 99.4% of predictions falling within Zones A and B of the Clarke Error Grid. This study demonstrates the potential of combining feature engineering and tree-based ML regression techniques for continuous glucose monitoring using wearable sensors.

## 1. Introduction

Recent advances in consumer wearable sensors have enabled continuous, real-time monitoring of physiological signals and behaviors in everyday settings. These sensors are portable, non-invasive, and increasingly affordable, making them promising tools for at-home health condition monitoring, early screening, disease management, and lifestyle interventions [1,2,3,4,5]. For example, the rich stream of physiological and biometric data captured by consumer wearable sensors, such as body movement patterns, heart rate, blood oxygen saturation, has already been successfully leveraged for applications including fall detection, frailty assessment [6], sleep apnea detection [7,8], and atrial fibrillation [9]. Building on this momentum, there is growing interest in utilizing wearable sensors for broader applications in continuous health monitoring, with the goal of supporting personalized healthcare and remote patient management [10].

Among various health indicators, blood glucose levels stand out as an important biometric due to their central role in metabolic health. According to the International Diabetes Federation, 537 million adults were living with diabetes in 2021, and this figure is projected to rise to 783 million by 2045 [11]. In addition, the number affected by prediabetes (PRED)—a precursor to type-2 diabetes (T2D)—is on the rise [12]. However, prediabetes often shows no obvious symptoms, leading to underdiagnosis [13] and poor management [14]. There is increasing evidence that continuous monitoring of glucose can aid in the early detection, prevention, and management of diabetes [15,16,17]. In addition, PRED and T2D are strongly influenced by lifestyle factors, including diet, exercise, and alcohol consumption [18]. Consequently, their prevention and reversal can be achieved through lifestyle changes [19]. Research shows that real-time glucose monitoring can promote positive behavioral changes that improve glucose regulation in diabetic patients [16], and may also encourage healthier behaviors among normoglycemic individuals by increasing awareness of daily glucose fluctuations [17].

Despite its potential, existing commercial methods for real-time continuous glucose monitoring have several barriers that limit their widespread adoption in everyday, non-clinical environments. There are two such methods: Self-monitoring of blood glucose (SMBG) and continuous glucose monitoring (CGM). SMBG involves finger-prick blood sampling. It is often painful and only provides snapshot measurements of blood glucose levels, making it impractical for continuous monitoring. On the other hand, CGM systems use microneedles to track interstitial glucose levels at regular intervals and are less invasive and more convenient than SMBG. However, they remain costly, require frequent sensor replacements, and may cause bleeding or skin irritations [20]. In addition, CGMs have limited accessibility, as most CGMs are FDA-cleared and covered by insurance only for diabetic patients with a medical prescription [21]. These limitations hinder its widespread use, especially among undiagnosed or asymptomatic individuals.

To address these limitations, recent research has focused on developing fully non-invasive and affordable glucose monitoring methods that can be safely used at home by everyone. Approaches involving optical, thermal, and electromagnetic sensing techniques have been explored over the past decade [22,23,24,25]. However, a major challenge is that other molecules and bodily fluids share similar properties, leading to interference, and making it difficult to achieve satisfying accuracy and reliability [26].

An emerging alternative is the use of multi-modal wearable sensors that collect data from multiple sources. Integrating signals from multiple modalities can help offset the limitations of individual sensing techniques. Wearable devices, including smartwatches and fitness trackers, enable real-time tracking of physiological signals and behaviors, such as heart rate, temperature, stress, and physical activity at high frequency. This multi-modal information can support continuous glucose estimation, given the reciprocal relationship between these factors and glucose regulation [27,28,29,30,31,32,33].

A few prior studies have applied machine learning (ML) techniques to uncover the complex dynamics of glucose fluctuations by combining data from multi-modal wearable sensors and manual user logs. These studies include hypo- and hyperglycemic event detection and prediction [34,35], post prandial glucose peak prediction [36], and glucose level prediction in normoglycemic and prediabetic populations [37,38,39,40]. However, many of these studies rely heavily on manual logging of food intake and activity [37,38].

In this study, we aim to address this limitation by developing computational models for non-invasive CGM using only passively sensed data, such as heart rate and temperature, that can be continuously collected from wearable devices in free-living conditions, without requiring active user input. The key contributions of this study are as follows:

- We developed continuous glucose prediction models using data that can be easily acquired with wearables in free-living conditions and investigated the effectiveness of various ML techniques.
- We systematically examined the feature importance and identified the feature categories that contribute most to model performance.
- We benchmarked our models against the state-of-the-art performance (SOAP) and demonstrated the superiority of our models.

## 2. Materials and Methods

### 2.1. Dataset

We utilized the BIG IDEAs Lab Glycemic Variability and Wearable Device Data (BIGIDEAs) dataset, which comprises data from 16 participants aged 35–65 with high-normal blood glucose (HbA1c 5.2–5.6) or prediabetes (HbA1c 5.7–6.4) [41]. This dataset was selected for our study due to the availability of simultaneous glucose readings and wearable data collected in minimally controlled settings. The same dataset has been used in several related studies on multi-modal glucose prediction [37,38], and it is publicly available on the PhysioNet platform [42]. Table 1 summarizes the demographic information and glucose metrics of the BIGIDEAs dataset.

Participants were recruited from the Duke Endocrinology Clinic via medical record review. Those with serious illnesses or those taking anti-diabetic medications were excluded. Data collection occurred over a period of 8–10 days, during which participants were provided with a standardized breakfast every other day. HbA1c levels were measured in the clinic to confirm that the levels fell within the range specified for the study.

Glucose data were collected using Dexcom G6 CGM systems (Dexcom, Inc., San Diego, CA, USA), which automatically record glucose levels (mg/dL) every 5 min. Physiological signals were measured using Empatica E4 wristbands (Empatica, Inc., Cambridge, MA, USA), which are equipped with four sensors: A photoplethysmography (PPG) sensor, an electrodermal activity (EDA) sensor, a skin temperature (sTemp) sensor, and a triaxial accelerometer. The PPG sensor records the blood volume pulse (BVP) signal at 64 Hz which is then used to calculate the heart rate (HR) at 1 Hz. EDA and sTemp are recorded at 4 Hz, while accelerometry data are recorded at 32 Hz. In total, approximately 26,000 glucose measurements, along with an equivalent duration of simultaneous wearable data were used in this study for developing the CGM models.

### 2.2. Preprocessing

The data preprocessing pipeline comprised four main steps: (1) dataset preparation, (2) outlier removal and filtering, (3) segmentation into epochs, and (4) missing value imputation.

(1) Dataset preparation
  As the first step, glucose values (mg/dL) recorded from the Dexcom G6 and signals recorded from the Empatica E4 were loaded from the dataset, along with their corresponding timestamps. Samples with missing timestamps were removed, and in cases of duplicate timestamps, only the first occurrence was retained. The tri-axial accelerometer data consisted of acceleration for x, y, and z axes. These values were used to compute the vector magnitude of acceleration (ACC) to represent the overall intensity of movement.
(2) Outlier removal and filtering
  This step was tailored to the characteristics of each signal. First, the BVP, EDA, and ACC signals were filtered in the frequency domain following established practices in the literature. The BVP signals were filtered using a fourth-order bandpass filter with a frequency range of 0.5–5 Hz. This frequency band helps remove motion artifacts, baseline wander, and noise due to environmental factors or sensor interferences, such as muscle or electrical noise [43]. For ACC signals, we employed a lowpass filter with a cutoff of 10 Hz to filter out noises introduced by mechanical vibrations, electrical interference, and environmental conditions [44]. The EDA signals were filtered using a low-pass filter, with the cutoff frequency set to 0.5 Hz, as the skin conductance signal is limited to this frequency [45,46]. Next, for each signal, only data within physiologically plausible ranges (HR: 25–240 bpm; sTemp: 30–40 °C; BVP: −500 to 500 (a.u.); EDA: 0.01 to 100 μS; ACC: 0 to 68 $m/s^{2}$) were retained; other values were deemed invalid and replaced with NaN.
(3) Segmentation into epochs
  The signals were synchronized and segmented into epochs using the timestamps from the Dexcom G6 and the Empatica E4. First, the epoch size for each signal was determined using the sampling frequency and the epoch duration. Subsequently, the signals were segmented into epochs and synchronized with the available glucose timestamps. As a result, each epoch represented the 5-minute window preceding a glucose reading. The number of data points in each epoch depended on the sampling rate of the signals. For example, HR data sampled at 1 Hz consisted of 300 data points per epoch, whereas EDA data sampled at 4 Hz consisted of 1200 data points and ACC data sampled at 32 Hz consisted of 9600 data points per epoch, respectively. Epochs with more than 50% missing values in at least one signal were discarded.
(4) Missing value imputation
  In the final step, missing values for each signal were imputed based on the epoch-wise distribution. If the distribution was approximately normal (defined as abs(skewness) < 0.5), the mean was used for imputation; otherwise, the median was used.

### 2.3. Feature Engineering

#### 2.3.1. Feature Construction

We conducted a rigorous feature engineering process to capture multiple domains of information, with the condition that the source signals for these features can be readily obtained in free-living conditions. Consequently, we excluded features that require active user input or clinical testing, such as food, activity, and sleep logs, clinical laboratory results, and long-term historical information extending beyond 2 h prior to prediction. The constructed set was organized into four primary categories: (1) physiological features, (2) behavioral features, (3) circadian features, and (4) demographic features. Each of these categories are explained in detail below.

(1) Physiological features
  Physiological features were included to capture changes in autonomic nervous system activity that can be both a response to and a trigger for glucose fluctuations. Physiological features were further divided into the following subcategories: (a) data-driven features, (b) HRV features, and (c) EDA tonic and phasic features.
  (a) Data-driven metrics
    The data-driven features included time, frequency, and non-linear domain features obtained from physiological signals.
    Time and frequency domain features are extensively used in biomedical signal processing to capture temporal trends and spectral characteristics of physiological signals [8]. On the other hand, non-linear features have been used in the literature to quantify complexity, irregularity, and deterministic patterns which cannot be adequately captured by solely relying on traditional time- and frequency-domain features [47]. Non-linear features can provide complementary information about underlying signal dynamics and improve model predictions by capturing subtle variations linked to physiological changes and transitions that precede or accompany changes in glucose levels. We constructed non-linear features including recurrence quantification analysis (RQA) features, entropy-based features, fractal features, and complexity-based features using EntropyHub, pyEntrp, Nolds, ordpy, and PyRQA libraries [48,49,50].
    We constructed 31 time-domain, 14 frequency-domain, and 42 non-linear features (a total of 87 data-driven features) for each of the HR and sTemp signals. Details of these features can be accessed at [51].
  (b) HRV metrics
    Following common practice in HRV signal analysis [43], we derived 13 HRV features from the preprocessed BVP signals to capture variations in heart rate and autonomic nervous system activity, such as RMSSD, SDSD, and pNN20.
  (c) EDA metrics
    The EDA signal is composed of a slow-varying tonic component related to the baseline level of skin conductance and a fast-varying phasic component related to sudden changes in skin conductance in response to stimuli. Following common practice in EDA signal analysis [46], we derived 42 features related to tonic and phasic components of the EDA signal, such as tonic_mean, tonic_std, tonic_energy, phasic_mean, phasic_std, and phasic energy.
(2) Behavioral features
  Behavioral features reflect the effects of lifestyle factors, particularly physical activity and eating, on glucose regulation through modulating insulin sensitivity and glucose uptake. Activity metrics related to the preceding 2-h window for each epoch were derived from the ACC data. Three behavioral features—ACC_2h_min, ACC_2h_max, and ACC_2h_mean—were computed.
(3) Circadian features
  Circadian features represent the influence of circadian rhythm on glucose metabolism, accounting for time-of-day variations in insulin sensitivity and glucose levels. Three circadian features were derived from the timestamps. The first feature, “minutes from midnight”, indicates the time of day. Subsequently, we applied sine and cosine transformations to this feature to account for the cyclical nature of the circadian rhythm.
(4) Demographic features
  Demographic features help account for inter-personal variability in baseline glucose dynamics. The biological sex was used to derive demographic features. One-hot encoding was applied to convert this categorical data into binary. Specifically, ‘male’ was mapped to ‘1’ and ‘female’ was mapped to ‘0’.

Feature cleaning involved removing features with more than 20% missing values, overflow or constant values, duplicates, and low-variance (variance < 1 $\times \;10^{-5}$). Subsequently, rows containing infinite values or more than 20% missing data were excluded.

#### 2.3.2. Feature Selection

**Figure 1:**
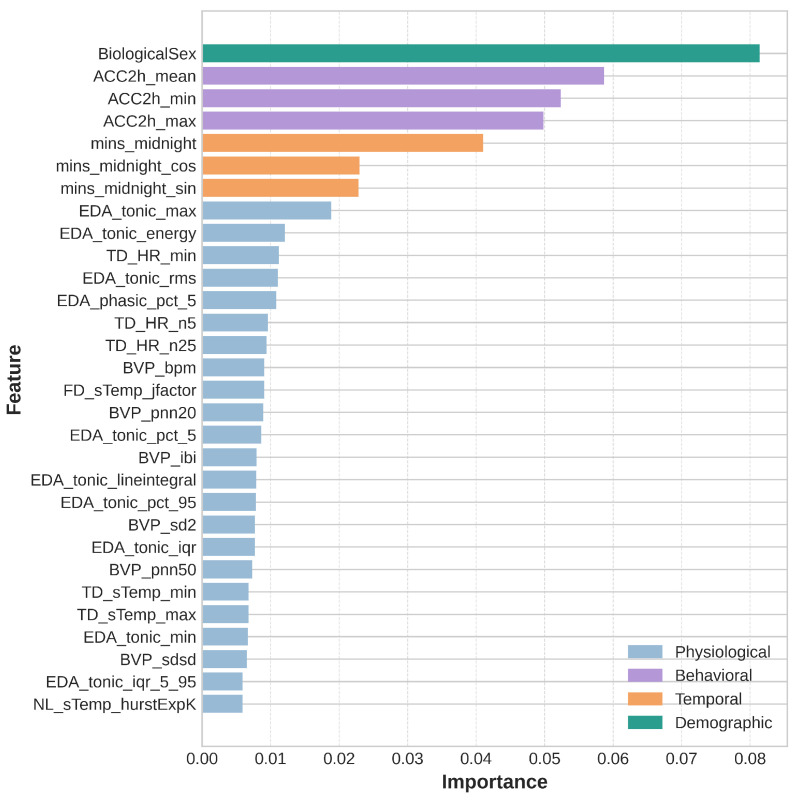
The 30 selected features in descending order of importance, color-coded based on the feature category.

The dataset was divided into an 80/20 train–test split, resulting in 21,104 epochs in the training set and 5276 epochs in the testing set. We then performed feature selection to identify a subset of the most important features among all the features extracted in the previous section. A smaller feature set is likely to speed up the model training process, prevent overfitting, and improve generalizability [52,53]. It is worth noting that only data from the training set was used to perform feature selection to avoid potential data leakage [54]. To determine feature importance, we used a random forest regression algorithm to generate importance scores for each feature and sorted them accordingly. The top 30 features were retained for model training (Figure 1).

### 2.4. Model Training and Validation

Following the feature selection process, the resulting dataset for model training consisted of 30 features as inputs and glucose measurements as the target variable.

We applied four ML regression algorithms: linear regression (LR), ridge regression (RR), random forest regression (RFR), and XGBoost regression (XR). Each algorithm was integrated into a pipeline that included an imputer, followed by a scaler and a regressor. The median was used to impute missing values, and standard scaling was applied to transform the features to a common scale with zero mean and unit variance, as some algorithms are sensitive to feature scales. Hyperparameter tuning was performed using grid search with 5-fold cross-validation.

### 2.5. Model Testing

The models were evaluated in terms of both prediction and clinical accuracy. For prediction accuracy, we employed performance measures including the coefficient of determination (R-squared), root mean squared error (RMSE), normalized root mean squared error (NRSME), and mean absolute relative difference (MARD). Higher R-squared values indicate better model performance, as they reflect a higher proportion of variance explained by the model. Conversely, lower errors indicate better performance, as they represent smaller deviations from the reference glucose values. It is worth noting that the model training, the validation and testing process was repeated 20 times under different random seeds. The average values of the performance measures across these 20 repetitions were used as the final performance metrics for each model. This approach provides a more robust statistical perspective on model performance.

In addition to the quantitative performance measures above, Bland–Altman (BA) plots were employed to assess the level of agreement between the predicted values and the reference glucose values [55,56]. These plots help identify systematic biases, detect trends in prediction, and assess the limits of agreement between the predicted and reference glucose values.

To complement the prediction accuracy analysis, we also evaluated the clinical accuracy of the models using the Clarke Error Grid (CEG), one of the golden standards for evaluating the clinical accuracy of glucose meters [57]. The CEG divides the scatterplot of predicted and reference glucose values into five regions based on the impact of the predictions on the risk of incorrect treatment. Predictions falling into zones A and B are considered safe and clinically acceptable, while those in zones C, D, and E indicate progressively higher risks of unnecessary treatment, missed detection of hypoglycemia or hyperglycemia, or confusion between treatments for the two conditions. To facilitate understanding, the regions were color-coded for better visualization: Zone A (green), Zone B (yellow), Zone C (orange), Zone D (purple), Zone E (pink).

**Figure 2:**
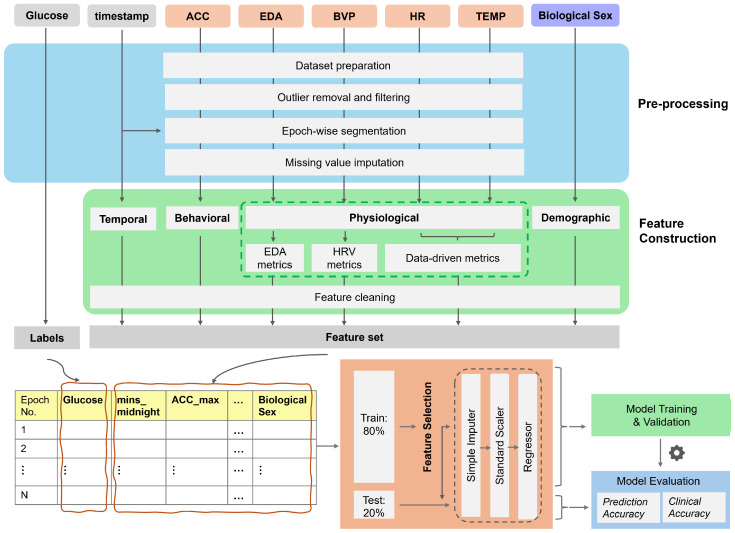
Overview of the methodology.

Figure 2 presents the overview of the methodology used in the study, outlining the pipeline from input sensor modalities through preprocessing, feature engineering, model training, and validation, to model evaluation.

## 3. Results

### 3.1. Prediction Accuracy

#### 3.1.1. Performance Metrics

Table 2 presents a summary of the model performance (mean ± standard deviation) across the 20 repetitions. The XR model shows the best performance with the highest R-squared, and lowest RMSE, NRMSE, and MARD. The RFR model also performed reasonably well, with comparable results to the XR model in terms of all the given metrics. In contrast, the two linear models (LR and RR) showed a tendency of underfitting, with low values for R-squared, and high values for RMSE, NRMSE, and MARD.

#### 3.1.2. Level of Agreement

**Figure 3:**
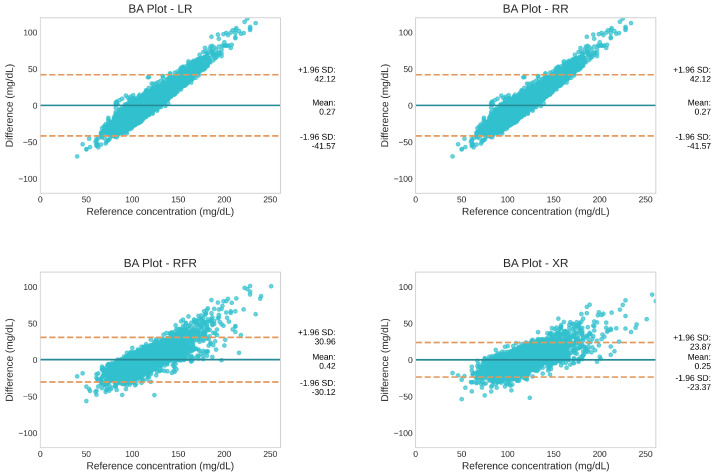
Comparison of the level of agreement between the reference and predicted glucose values using BA plots for the four regression models. The mean difference (MD) is represented by the solid blue line, and the upper and lower limits of agreement (LOAs) are represented by the two dotted orange lines.

Figure 3 shows the BA plot comparing the predicted glucose values with the reference values for the four models for one of the train–test splits. The x-axis represents the reference values and the y-axis represents the difference between the reference and predicted values. The solid blue line represents the mean difference (MD) and the two dotted orange lines represent the upper and lower limits of agreement (LOAs). The LOAs are computed as MD±1.96×SD, where SD is the standard deviation of the differences. A mean difference close to zero indicates that there is no significant systematic bias. A narrow range of the LOA is preferred, which indicates a high level of agreement between the reference and predicted values.

All four models exhibited an MD close to zero, indicating no systematic biases. Among the models, the XR model had the smallest mean difference of 0.25 mg/dL. When comparing LOAs, tree-based models outperformed linear models by a large margin, showing significantly narrower LOAs than the linear models. Moreover, a noticeable trend of overestimation in the hypoglycemic region and underestimation in the hyperglycemic region was evident in both linear models. However, this trend was less pronounced in the RFR model and was further diminished in the XR model.

### 3.2. Clinical Accuracy

**Figure 4:**
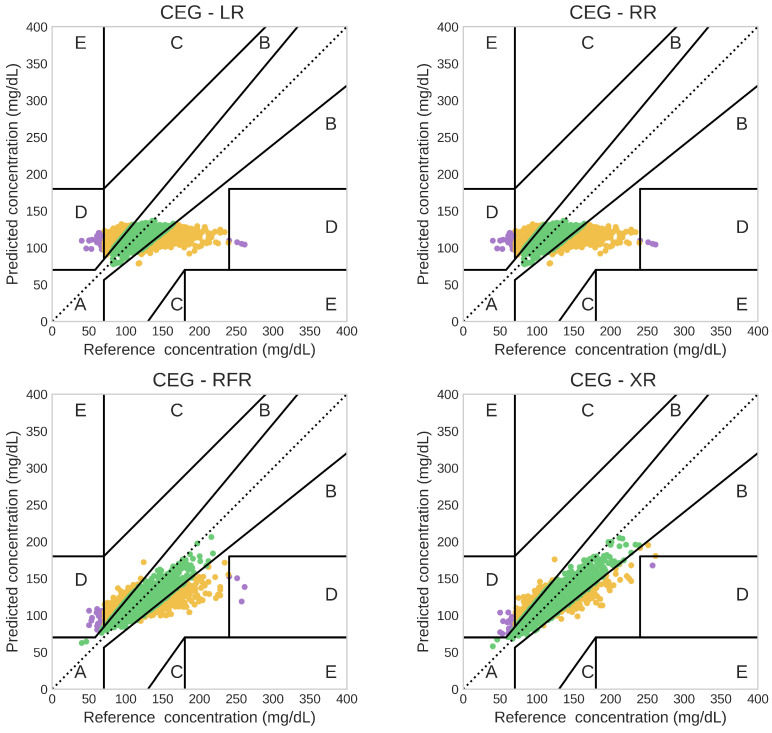
Comparison of the clinical accuracy of predictions based on the Clarke Error Grid (CEG) for the four regression models. Letters A–E correspond to the five CEG zones and are color-coded as follows: A (green), B (yellow), C (orange), D (purple), and E (pink).

Table 3 shows the percentage of predictions that fell into each zone of the CEG for the four regression models in one of the train–test splits, and Figure 4 presents the corresponding CEG plots.

More than 99% of predictions of all four models fell into Zones (A and B) of the CEG. However, out of these 99%, only around 77% of predictions were in Zone A for the LR and RR models, whereas around 90% and 95% of predictions were in Zone A for RFR and XR, respectively. None of the predictions fell into Zones C and E in any of the four models. A small percentage of predictions of each model (0.6–0.8%) landed in Zone D, indicating a failure to detect hypoglycemia or hyperglycemia in a few instances.

### 3.3. SHAP Explanations

To obtain a more transparent understanding of how each input feature contributed to the predictions, we utilized Shapley Additive exPlanations (SHAP) [58]. A SHAP summary plot was generated for the best-performing XR model, where the x-axis represents the SHAP values, while the y-axis lists the features ranked by their overall importance. Each dot corresponds to an individual data point, and is color-coded based on the feature value. This helps visualize both the magnitude and direction of the impact of each feature on the predicted glucose levels.

**Figure 5:**
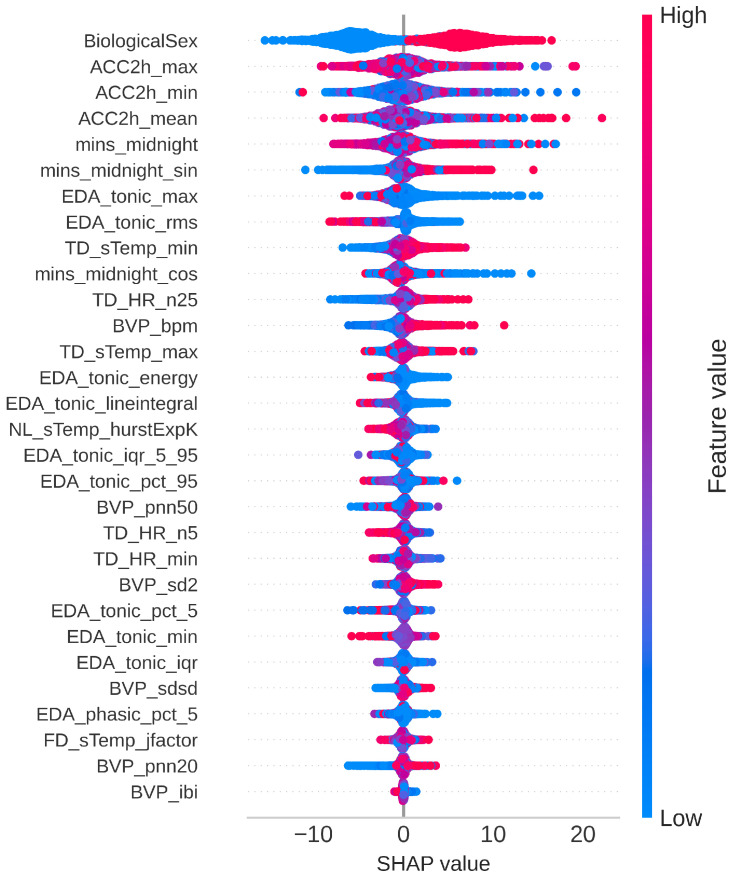
SHAP summary plot for input features of the XR model.

For several features, high and low feature values, represented by contrasting colors, were associated with SHAP values of opposite signs, highlighting the directionality of their effect on the model output (Figure 5). For instance, for ‘BiologicalSex’, the lower values (‘0’) denoting ‘female’ were linked to a decrease in the estimated glucose reading, whereas the higher values had the opposite effect. A similar trend was observed for ‘mins_midnight_sin’, and ‘TD_sTemp_min’. In contrast, higher feature values for ‘EDA_tonic_rms’ were associated with a decrease in the predicted glucose level, and vice versa.

## 4. Discussion

In this study, we developed continuous glucose prediction models using machine learning techniques and data that can be easily obtained in free-living environments. We identified key features and conducted a comprehensive evaluation of both the prediction and clinical accuracy of the models. In the following discussion, we place our findings in the context of existing literature and highlight their implications.

### 4.1. Feature Importance

Our feature selection and post hoc SHAP analysis revealed several key insights into the features contributing to glucose prediction. One of the most interesting findings was that biological sex emerged as the top feature. This aligns with previous studies documenting inherent differences in glucose regulation between men and women [31]. In addition to sex, behavioral features also ranked among the top five and exhibited the high absolute SHAP values. This is likely due to their strong association with activities that directly impact glucose levels, such as eating and exercising. Circadian features also played a significant role, with all three circadian-related features appearing among the top selected features. This echoes the established understanding that circadian rhythms play an important role in metabolic processes and glucose homeostasis [30].

Physiological features, particularly those derived from the tonic components of the EDA signal, dominated the feature set. Ten out of the 23 selected physiological features came from the EDA tonic features, suggesting that the baseline level of skin conductance is an important predictor of glucose levels. Based on the SHAP plot, the predicted glucose level decreased when EDA_tonic_rms values were high, and vice versa. A physiological basis for this could be increased sympathetic nervous system activity, which can enhance glucose uptake by skeletal muscles during stress or arousal, or reflect hypoglycemic symptoms such as sweating—both of which are associated with lower glucose levels. An association between the elevated tonic component of the EDA and hypoglycemia was also reported in a previous study [35].

### 4.2. Model Performance

In terms of model performance, tree-based models outperformed the linear models. This aligns with existing literature, where RFR and XR have consistently demonstrated superior performance in glucose prediction tasks [37,38]. Among these, the boosting-based XR model emerged as the best-performing model. The two linear models showed similar performance, and regularization in the RR model did not yield a substantial improvement. This suggests that linear models may be insufficient for capturing the complex, non-linear dynamics involved in glucose regulation.

Focusing on the XR model, it achieved an R-squared value of 0.73, indicating that it could explain approximately 73% of the variance in glucose levels. This substantial predictive power is further validated by low RMSE and NRMSE values. In addition, the MARD of 7.1% falls well below the expected range for commercial CGM systems, which typically report MARD values between 9 and 16% [20].

However, despite these promising results, the XR model exhibited a slight tendency to overestimate glucose values in the hypoglycemic region and underestimate them in the hyperglycemic region, as depicted by the BA plot in Figure 3. This discrepancy may be attributed to the distribution of the training data, which included predominantly normoglycemic and prediabetic individuals. Consequently, extreme glycemic events were underrepresented in the dataset, which could limit the model’s accuracy in predicting values in these regions.

Nevertheless, the XR model demonstrated strong clinical accuracy. Approximately 95% of its predictions fell into Zone A of the CEG, indicating clinically acceptable predictions. Furthermore, more than 99% of the predictions fell into Zones A and B, aligning with the clinical accuracy criteria outlined in ISO 15197:2013 for SMBG devices [59].

### 4.3. Comparison with Prior Work

There have been a few pioneer studies on continuous glucose prediction using wearable data. However, this study has several advantages. First, we intentionally avoided the use of food logs, activity logs, and clinical metrics to ensure that the developed model could be readily integrated into existing consumer wearable devices, where such detailed information might not be available. Second, to address the common concerns of using ML methods in clinical applications, we selected shallow ML rather than deep learning techniques. As a result, our models are less computationally expensive, making them more suitable for real-time applications. Furthermore, our models provide greater transparency in domain-specific feature construction and selection.

Table 4 compares the results of the best model with those of related studies. Since R-squared and NRMSE values were not reported in most studies, these metrics are not included in the comparison. Our XR model achieved an RMSE of 11.9 mg/dL and an MARD of 7.1%, outperforming the state-of-the-art model developed in [38]. Our model’s performance is also comparable to the RF model developed in [37]. However, since both studies did not include an evaluation of clinical accuracy, a direct comparison in this regard was not possible. In contrast to these two studies, our model was developed without relying on additional data that are typically used in controlled settings but are often unavailable in free-living conditions. In addition, our model performed favorably when compared to the deep-learning-based BiLSTM model [39] both in terms of prediction accuracy and clinical accuracy.

### 4.4. Clinical Applicability

The tree-based models demonstrate strong prediction and clinical accuracy, and they also hold promising applicability in clinical settings. They incorporate a broad range of features that account for physical activity, stress, and other physiological changes, which are known to significantly affect glucose levels. However, the models do not include diet-related data, which may limit their ability to capture rapid glucose fluctuations, such as those following the consumption of high glycemic index (GI) foods. This exclusion may be a constraint in detecting certain glucose dynamics, especially those linked to dietary intake. Nonetheless, the design of the models remains focused on factors that can be easily monitored, which potentially enhances their practical use in real-world, free-living conditions. It is also important to note that the trends shown in the BA plot may have significant clinical implications when managing extreme glycemic events. For instance, relying on linear models could potentially delay timely intervention during hypoglycemic episodes due to the tendency to overestimate glucose levels in this range. Conversely, underestimation in the hyperglycemic range could result in prolonged exposure to elevated glucose levels, increasing the risk of long-term complications. Therefore, tree-based models not only have better performance metrics, but also offer improved clinical applicability.

### 4.5. Limitations

Despite the promising results, the current approach comes with a few limitations. First, the study was based on data from normoglycemic or prediabetic individuals, with limited representation of extreme glycemic conditions. This could significantly limit the model’s generalizability to broader populations. Next, the ground truth values for blood glucose were obtained using a commercial CGM sensor. However, there are instances when interstitial glucose levels may deviate from blood glucose levels, particularly during periods of rapid fluctuations [60]. Nonetheless, there is no better alternative for obtaining the ground truth in large multi-modal datasets, as using SMBG devices, which directly measure blood glucose, is highly impractical due to the need for frequent finger-pricking. Finally, the use of historical data from the accelerometer within a 2-h window prior to estimation could pose challenges to continuous, real-time glucose prediction. Users may remove wearables during showers or for charging, which could lead to missing data. If significant portions of this crucial 2-h window are missing, it could affect the model’s ability to make accurate predictions, especially for users with irregular wear time.

### 4.6. Future Work

Future work should focus on testing the external validity of the models on additional datasets from larger, and more diverse cohorts to address the current limitation in participant diversity. In addition, future work could explore integrating chemical biomarkers, such as sweat glucose, alongside physiological signals and behaviors. The inclusion of chemical biomarkers could provide a more direct measure of glucose dynamics, enhancing both the biological relevance and predictive accuracy of non-invasive glucose prediction models.

## 5. Conclusions

In this study, we developed non-invasive continuous glucose prediction models using wearable data and demographics. The top features aligned with established knowledge of the reciprocal relationship between glucose regulation and related factors. Linear regression techniques were found to be inadequate to capture complex glucose dynamics, whereas tree-based methods were found to be appropriate. The performance of the best model (XR) was comparable to the state of the art and within the standards for commercial CGM and SMBG systems, despite not using additional manually logged data. Our modeling process also offers several advantages compared to the current state of the art. It is simpler, less computationally expensive, and more transparent. Moreover, the models are fully automated without the need for manual input. Future work should focus on validating the models on external datasets before they can be adopted for commercial use.

## Figures and Tables

**Table 1:** Demographic information and glucose metrics.

Item	Categories	Value
Number of subjects		16
Age range		35–65 years
Gender	Male	7 (43.75%)
	Female	9 (56.25%)
HbA1c		5.7 ± 0.3
Glucose Metrics ^1^	Average glucose	115 mg/dL
	Time in range (TIR) ^2^	97.87%
	Time above range (TAR) ^3^	1.57%
	Time below range (TBR) ^4^	0.56%
No. of epochs ^1^		26,380

^1^ Values computed post preprocessing; ^2^ The percentage of time within 70–180 mg/dL; ^3^ The percentage of time above 180 mg/dL; ^4^ The percentage of time below 70 mg/dL.

**Table 2:** Comparison of model performance.

Model Name	R-Squared	RMSE (mg/dL)	NRMSE (mg/dL)	MARD (%)
LR	0.13 ± 0.01	21.3 ± 0.3	0.93 ± 0.00	13.7 ± 0.1
RR	0.13 ± 0.01	21.3 ± 0.3	0.93 ± 0.00	13.7 ± 0.1
RFR	0.53 ± 0.01	15.6 ± 0.3	0.68 ± 0.01	9.7 ± 0.1
XR	0.73 ± 0.01	11.9 ± 0.3	0.52 ± 0.01	7.1 ± 0.1

**Table 3:** Comparison of the percentage of predictions in each zone of the Clarke Error Grid (CEG).

Model Name	Zone A (%)	Zone B (%)	Zone C (%)	Zone D (%)	Zone E (%)	Zones (A + B) (%)
LR	77.0	22.2	0.0	0.8	0.0	99.2
RR	77.0	22.2	0.0	0.8	0.0	99.2
RFR	89.1	10.3	0.0	0.6	0.0	99.4
XR	94.2	5.2	0.0	0.6	0.0	99.4

**Table 4:** Comparison of the best model with the state of the art.

Study	Best Model	RMSE (mg/dL)	MARD (%)	CEG Zones (A + B) (%)
Bent et al. [38]	XR	21.1	13.3	-
Ali et al. [37]	RFR	9.0	4.7	-
Huang et al. [39]	BiLSTM	13.4	12.0	97.0
This study	XR	11.9	7.1	99.4

## Data Availability

The BIG IDEAs Lab Glycemic Variability and Wearable Device Data dataset used in this study is publicly accessible at: https://physionet.org/content/big-ideas-glycemic-wearable/1.1.2/ (accessed on 21 April 2025).
